# Bioarchaeological evidence of decapitation from Pacopampa in the northern Peruvian highlands

**DOI:** 10.1371/journal.pone.0210458

**Published:** 2019-01-08

**Authors:** Tomohito Nagaoka, Mai Takigami, Yuji Seki, Kazuhiro Uzawa, Diana Alemán Paredes, Percy Santiago Andía Roldán, Daniel Morales Chocano

**Affiliations:** 1 Department of Anatomy, St. Marianna University School of Medicine, Kawasaki, Japan; 2 Yamagata University, Yamagata, Japan; 3 National Museum of Ethnology, Suita, Japan; 4 Faculty of Human Sciences, University of East Asia, Shimonoseki, Japan; 5 Universidad Nacional Mayor de San Marcos, Lima, Peru; Seoul National University College of Medicine, REPUBLIC OF KOREA

## Abstract

Little is known about the precise date of the emergence of decapitation in a ritual context and the presence of systematic postmortem modification patterns in the ancient Central Andes. The ceremonial complex at Pacopampa in the northern Peruvian highlands provides early osteological evidence of decapitation in six individuals dating to the latter half of the Late–Final Formative Periods (500–50 BC) and to the Early Cajamarca Period (AD 200–450). Based on osteological evidence, and when taken together with archaeological settings and settlement patterns, researchers can be certain that those whose heads were disembodied were not likely to have been involved in organized battles. In addition, the similarities in the cut-mark distribution, direction, and cross-sectional morphology of each individual's remains, as well as the characteristics of selected individuals, imply that the decapitated individuals were carefully prepared using a standardized method and that those who modified the heads may have been professional decapitators. This study offers indisputable bioarchaeological evidence of ritualistic offerings of human skulls and systematic postmortem modification patterns, which is consistent with a contemporaneous iconographic motif of decapitation and extends the chronology of this practice back to the Formative Period in the northern Peruvian highlands.

## Introduction

The Amerindian practice of taking and displaying trophy heads can be traced back thousands of years and was considered to have been associated with materialistic factors (e.g., territorial disputes over access to natural resources) and nonmaterialistic ones (e.g., a punishment of an individual in the afterlife, the establishment and reinforcement of social status, and the desire to obtain supernatural powers) [1].

In the ancient Central Andes, the taking and displaying of trophy heads was carried out by decapitating heads from the bodies and was often connected to the act of human sacrifice [2], an ultimate means of communication between humans and supernatural beings [3]. Andean people believed that the worldly difficulties they faced could be overcome through sacrifice, and decapitation was a frequent motif in their iconography [2]. Archaeological remains show evidence of different types of sacrifice: offering sacrifice (supplication on behalf of a living thing or an inanimate object to the supernatural), foundation sacrifice (act of dedication with offerings for ritual architecture), and retainer sacrifice (high-status funerary practices) [4]. Many ritual deaths may fall into one or more of these three conceptual categories [4]. Andean people believed that inanimate beings also contained life, so the offerings involved not only humans and animals but also inanimate objects [2]. The Spanish documented the sacrificial practices of the Inca (AD 1470–1532), which has aided immensely in understanding the archaeology of human sacrifice; however, these Incan rituals may not be representative of the practices of human sacrifice that occurred in preceding time periods [4]. The oldest evidence for human sacrifice in the Central Andes appears to be from the burials of children at La Paloma (5000–2800 BC) [5] and Ancón (400 BC) [6] on the central Peruvian coast and Alto Huallaga (700–250 BC) [7] in the northern Peruvian highlands. Such offerings may have been performed as part of building dedication rituals; however, prior studies on these sacrifices did not include any bioarchaeological descriptions of the excavated human remains, and the victims were not decapitated. It is thus possible these remains were originated from naturally deceased individuals and the motivation of the sacrifice was not elucidated.

Osteological proof of the oldest decapitation was potentially discovered among two headless adults and seven isolated non-adult and adult crania from Asia Beach (1300–1100 BC) [8] on the central Peruvian coast, and among five female crania from the site of Wichqana (1150–750 BC) [9] in the southern highlands. There are also a few cases of the ritual offerings of human skulls at the sites of Chavín (860–460 BC) [10, 11] and Kotosh (700–250 BC) [12] in the northern highlands. Some of these remains include possible decapitated crania with cut marks on the cranial vault and face, which were likely caused during the decapitation [9]. According to archaeological artifacts from the same time period, trophy heads were abstractly depicted on vessels and monoliths found at the ceremonial monuments discovered at Pacopampa [13], Huacaloma [14], and Kuntur Wasi [15] in the northern highlands. Additionally, a ceramic bottle depicting an individual who had slit his own throat was discovered on the northern coast, dating to the time of Cupisnique (1500–650 BC) [16].

The Nasca (200 BC–AD 750), Moche (AD 100–800), and Wari (AD 600–1000) societies, who flourished in the Early Intermediate Period and Middle Horizon, experienced increasing sociopolitical complexity and militarism compared to preceding years. These societies practiced decapitation, and a wealth of evidence supporting this practice has been documented. For instance, the trophy heads found at the Nasca drainage on the southern coast, which are interpreted as symbolic of regeneration, rebirth, and agricultural fertility, represent the largest collection of such remains from the Central Andes [17]. As for the identity and origin of the individuals transformed into trophy heads, the isotopic data suggested that the Nasca trophy heads were derived from the local Nasca population rather than enemy combatants [18].

The least disputable evidence of decapitation in the Central Andes hails from the Moche sites. The Moche iconography, known as the Presentation Theme, depicts the capturing of prisoners, mutilations, sacrifices, and dismemberments; the conflict was highly ritualized and aimed to capture enemies for sacrificial purposes rather than to conquer territories [19]. The excavation of the Pyramids of the Moon (Huaca de la Luna) on the northern coast uncovered human skeletal remains, which paralleled iconography in which specific individuals were subject to throat-slitting and decapitation among other types of bodily mutilation and manipulation as part of a ritualized postmortem treatment [20, 21]. Biodistance analysis of dental measurements suggest that the Moche sacrificial victims were foreign warriors captured during territorial battles with competing polities [22], although some archaeologists think that the victims were elites defeated in ritual battle against other elites from the same territory [20, 23]. Isotopic data support the gradual change in the practice from local- to foreign-origin individuals [24].

Similarly, in the Wari society, captives were taken during battles and raids and were decapitated for ritual purposes [25]. The majority of the trophy heads from Conchopata in the Wari heartland consisted of adult males with cranial trauma, indicating frequent involvement in violence [26, 27]. The results of the strontium isotope analyses of five of the crania revealed that some of the trophy heads consumed foods grown in nonlocal areas outside of the Wari heartland, suggesting that they were taken from foreigners [26, 27].

The multiple lines of evidence discussed above reveal the potential for decapitation in the Central Andes, at least during the Formative Period. Evidence of perimortem trauma consistent with ritual violence first appeared in this area during the Middle and Late Formative Periods (1200–250 BC), after the time period when building dedication rituals would have been practiced. Additionally, decapitation may have appeared in the highlands in the latter half of the Late Formative Period (500–250 BC) and the Final Formative Period (250–50 BC), later than on the coast. However, the precise date of the emergence of decapitation in a ritual context, as well as the presence of systematic postmortem modification patterns, remains unknown, as most studies have not provided bioarchaeological description of decapitation. Using data with a known archaeological context, the ceremonial complex at Pacopampa provides early osteological evidence of decapitation in a ritual setting in the northern Peruvian highlands, which can aid in testing the hypothesis that the preferable iconographic depiction of trophy heads represented reality in the Formative Period of the Central Andes. Here, the term “trophy heads” refers only to those with postmortem modification [28].

### Archaeological setting of Pacopampa

The Formative Period involved numerous cultural changes, including the advent of sedentism and social complexity [29]. The first construction of public architecture in the Central Andes dates to about 3000 BC, a time that some scholars define as the beginning of the Formative Period [29]. The Middle and Late Formative Periods (1200–250 BC), at least in the northern highlands, are further characterized by an impressive array of ceremonial architecture including platforms, plazas, and decorated stone sculptures, which was continually rebuilt and renovated through collaborative efforts, as well as social complexity and a lack of political control over surplus agricultural products [30].

**Fig 1:**
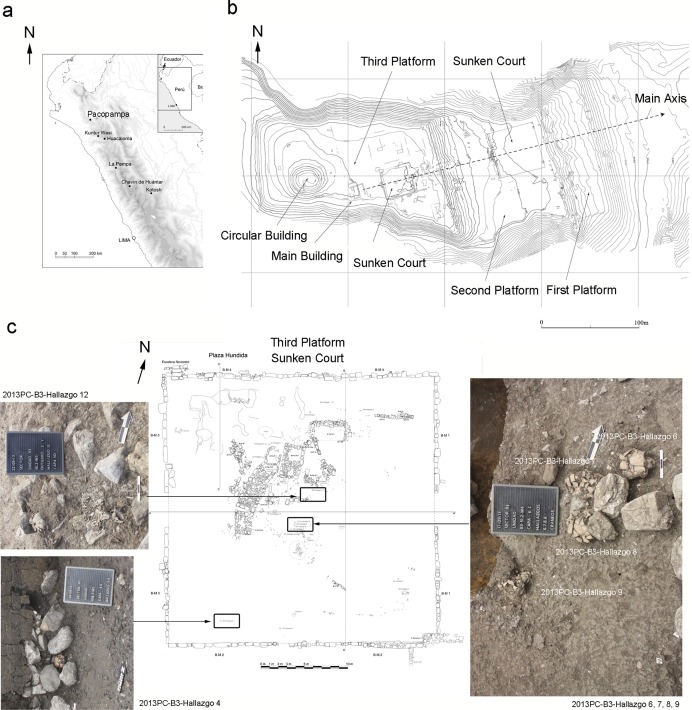
Map. (a) Map of Peru showing the location of Pacopampa. (b) Map of the Ceremonial Complex at Pacopampa. (c) Map of the sunken court of the third platform.

Pacopampa is one of the largest Formative Period sites in the northern highlands of Peru, located at an altitude of 2,500 m (Fig 1A). It is roughly 70 km from the Pacific coast. Rafael Larco Hoyle, a Peruvian archaeologist, conducted the first excavations at Pacopampa in 1939. The variety of foreign artifacts including shellfish objects, obsidian, and cinnabar recovered from Pacopampa reveals the wide-ranging connections formed by the ceremonial center and the patterns of exchange that were essential to maintaining the prestige of Pacopampa and its elites [31]. The Pacopampa site was the ceremonial center in the northern highlands, which is evidenced by a large-scale public architecture and plaza, decorated stone sculptures, several high-status burials, and archaeological remains not related to subsistence (e.g., tools for preparing hallucinogenic snuff) [31]. Prior studies on such artifacts revealed that Pacopampa was established almost contemporaneously with the Chavín de Huantar site, which is representative of the cult culture that occurred during this phase.

The Pacopampa Archaeological Project, directed by Yuji Seki and Daniel Morales, performed excavations at the site from 2005 to 2017. During the Middle and earlier half of the Late Formative Periods, three low platforms surrounding a sunken plaza were constructed at Pacopampa; these constructions may have been the most important religious space in the overall complex (Fig 1A). In one case, an unusual human skeleton dating to the beginning of the Pacopampa II cultural phase (the earlier half of the Late Formative Period, 800–500 BC) was recovered at the third platform with a pair of gold earplugs, a pair of gold earrings, and shell objects [31]. A detailed examination of the neurocranium revealed the presence of a fronto-occipital type of artificial cranial deformation, which had not previously been found among lower-class human remains [32]. The precious goods found in their graves and their special burial treatment demonstrates that social stratification at Pacopampa may date back to the beginning of the Pacopampa II cultural phase.

The excavation of the third platform, where core ritual architecture was concentrated, revealed that the main buildings were associated with the sunken court and were rebuilt and renovated during the Pacopampa II cultural phase. The Pacopampa Archaeological Project further focused on the excavation of the sunken court and unearthed smaller structures, such as platforms and rooms inside the court. They belonged to a post-Formative occupation, which is characterized by the covering up of the original architecture without destroying it during the Early Cajamarca Period (AD 200–450) (Fig 1B and 1C). The offering dedications contain six isolated human crania, of which the crania, mandibles, and cervical vertebrae remained articulated when they were excavated (Fig 1C), as well as miniature vessels, small gold accessories and sheets, copper needles, stone beads, a quartz and bone awl, and stone points; all of this points to the idea that the pieces had been intentionally deposited, suggesting a form of complex ritual activities or practices. Four out of six isolated crania were located close to each other and were facing to the east (individual numbers 2013PC–B3–Hallazgo 6, 7, 8, and 9) (Fig 1C). However, the direction of the remaining two (individual numbers 2013PC–B3–Hallazgo 4 and 12) was difficult to be determined due to poor preservation. After placing the offerings, the sunken court was immediately sealed with thick layers of clay and stones by the people of the Early Cajamarca Period. It is possible that the offerings around the structures, as well as the sealing of the sunken court, constituted a series of ritual activities during the Early Cajamarca Period; the isolated crania were likely to have been an important part of such rituals. These specimens are early osteological evidence of decapitation in the northern Peruvian highlands and could be instrumental in refining our understanding of the ritual violent behaviors of the ancient Andean people. In the context of this paper, violence refers to direct action intended to harm or kill another person. Violence-related skeletal trauma visible in archeological remains often has been linked to warfare, domestic violence, corporal punishment, and physical conflict resolution, as well as ritual activities in the Andes [33]. Violence can be thus interpreted as a destructive act (e.g., harming or killing to eliminate enemies) or a constructive act (e.g., ritual killing as an offering to the supernatural). The purpose of this study is to provide bioarchaeological descriptions of the decapitated remains and to explore variations in ritual violent behaviors over time.

## Materials and methods

### Human remains

In total, twelve field seasons have yielded the recovery of human remains from 128 individuals. Through a combination of classification of archaeological artifacts and architecture styles and stratigraphy, it has been determined that the human remains belonged to three different cultural phases: (1) the Middle Formative Period (Pacopampa I, 1200–800 BC); (2) the earlier half of the Late Formative Period (Pacopampa II, 800–500 BC); and (3) the Early Cajamarca Period (AD 200–450). The total numbers of individuals from each of the three phases are 6, 97, and 25, respectively (S1 Table). In this study, we examined six isolated crania from the sunken court from the third phase.

The radiocarbon dating of the bones was performed by Beta Analytic, a laboratory in Miami, Florida and the IntCal13 dataset was used to calibrate the radiocarbon ages [34].

The materials are maintained by the Pacopampa Archaeological Project and are temporally housed at the Center for Pacopampa Archaeological Project (Jr. Bolognesi, Centro Poblado de Pacopampa, Distrito de Querocoto, Provincia de Chota, Region Cajamarca, Peru) under the permission of the Peruvian Ministry of Culture. All necessary permits were obtained for the described study, which complied with all relevant regulations: the permissions of the National Institute of Culture of Peru from 2005 to 2010 (Permission Nos: 1108/INC, 1868/INC, 979/INC, 1061/INC, 815/INC, and 1403/INC) and those of the Peruvian Ministry of Culture from 2011 to 2015 (Permission Nos: 265-2011-DGPC-VMPCIC/MC, 593-2012-DGPC-VMPCIC/MC, 006-2013-DGPA-VMPCIC/MC, 363-2014-DGPA-VMPCIC/MC, and 270-2015-DGPA-VMPCIC/MC). No aspect of the materials or methods of this study needed to be approved by our institutions’ ethical committees. This study was conducted according to the Vermillion Accord on Human Remains (approved by the World Archaeological Congress in 1989).

### Age and sex estimation

The ages of the crania were determined based on the formation of all crowns and roots and the eruption of each tooth [35], the degree of development and the closure of the occipital synchondrosis [36], and dental wear [37]. Adults who were 15 years or older at the time of their death were then classified into three categories: young (15–34 years); middle-aged (35–54 years); and elderly (≥55 years) [38]. The sex of the individuals over the age of 15 was determined based on a macroscopic assessment of nuchal crest, mastoid process, supra-orbital margin, supra-orbital ridge, and mental eminence [39].

### Examination criteria for trauma

The skeletal location, shape, diameter, and direction of the perimortem trauma were observed by the naked eye and meticulously recorded; trauma was diagnosed by the absence of new bone formations to distinguish between antemortem and perimortem trauma; additionally, the freshness of the broken surfaces (stains and colors) was examined to distinguish between perimortem trauma and taphonomic changes. Further classification then separated the trauma into wounds caused by metal versus stone tools using a cross-section morphology under both macroscopic assessment criteria [40] and microscopic assessment criteria [41–44]. Microscopic appearance is one of the most useful diagnostic characteristics in identifying cut marks on archaeological bones. Shipman and her colleagues suggested a diagnostic key to microscopic features distinguishing cut marks from marks resulting from various taphonomic events [41–44]. For microscopic observation, replicas of the bones were made using two procedures: a negative impression, or mold, was created from a silicone-based dental impression material, and a positive cast was created from an epoxy resin poured into the mold [45]. The products used for these procedures include a Provil Novo Light and Provil Novo Putty (Heraeus Kulzer, Germany) to make the molds and Stycast 1266 A/B (Emerson and Cuming, USA) to make the casts. The replicas were covered with an osmium plasma coating (NL-OPC80, Nippon Laser and Electronics Lab, Japan) and were then examined with a scanning electron microscopy (S4800, Hitachi, Japan) at the St. Marianna University School of Medicine.

Antemortem trauma was only examined macroscopically. Radiographic analyses were not available because the obtained permissions did not allow us to transport skeletal remains to our institutions for analyses. The skeletal locations, types (blunt or sharp force fractures) [46], mechanisms (direct or indirect types of fractures) [47], healing processes (inflammatory, reparative, or remodeling phases) [48], and complications of fracture (delayed healing, pseudoarthrosis, poor alignment, bone shortening, osteomyelitis, or avascular necrosis of bone) [47] were all considered for the purpose of describing trauma in detail and exploring the presence of healing reactions.

### Availability of materials and data

Those who wish to access the materials may do so by contacting the Pacopampa Archaeological Project.

## Results

Perimortem trauma was detected on five isolated crania (individual numbers 2013PC–B3–Hallazgo 4, 6, 7, 8, and 9), while antemortem trauma was detected on the remaining one isolated cranium (individual number 2013PC–B3–Hallazgo 12). Individual numbers 2013PC–B3–Hallazgo 4, 6, 7, 8, 9, and 12 were abbreviated as Hallazgo 4, 6, 7, 8, 9, and 12, respectively. The radiocarbon dating of five individuals revealed that they corresponded to two time periods: four individuals (Hallazgo 6, 7, 8, and 9) that were located in close proximity to one another belonged to 2140–2170 ^14^C yr BP, the latter half of the Late Formative Period (500–250 BC) and the Final Formative Period (250–50 BC), while one individual (Hallazgo 12) belonged to 1720 ^14^C yr BP, the Early Cajamarca Period (AD 200–450) (S2 Table). The six isolated crania were estimated to be male and from young to middle-aged adults (S3 Table).

### Perimortem trauma

**Fig 2:**
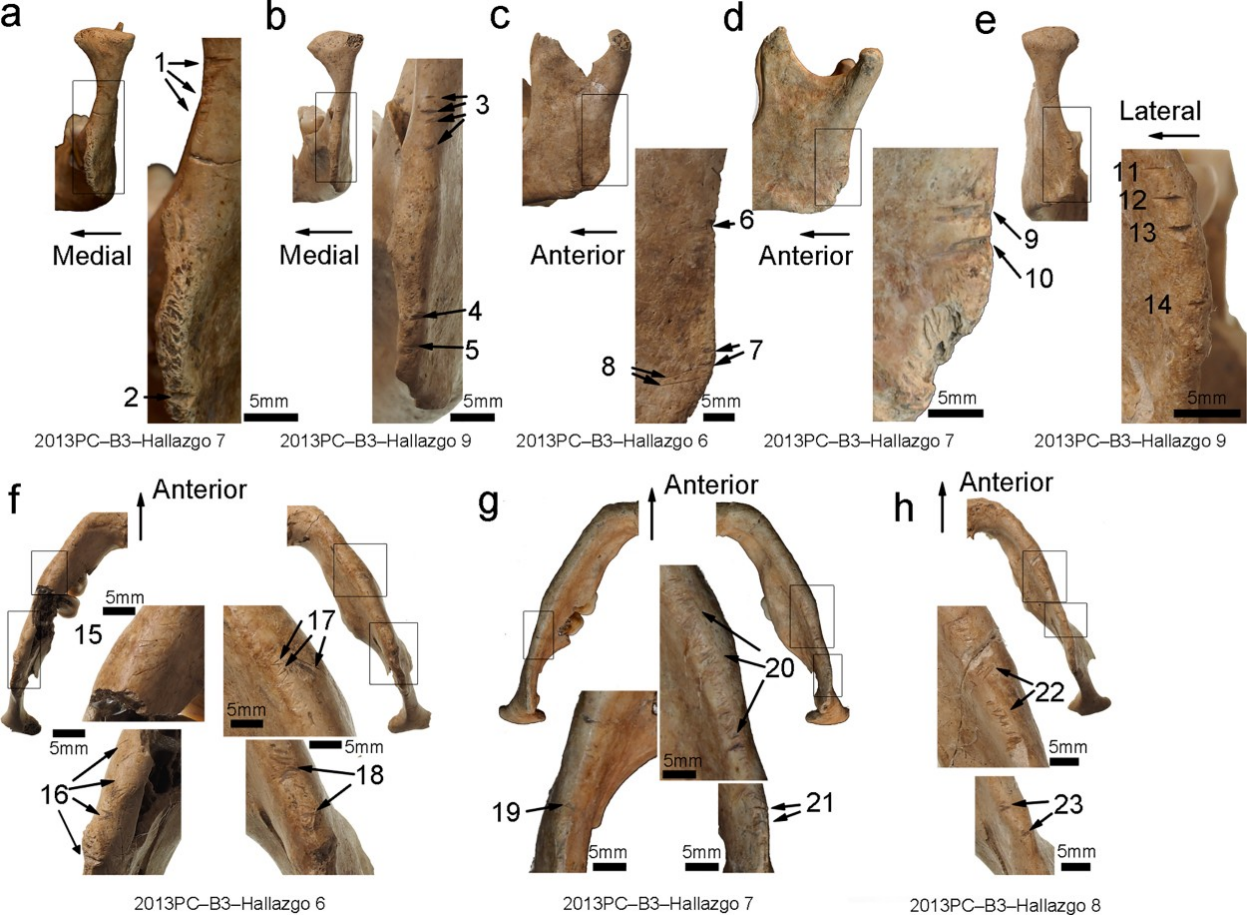
Macroscopic observation of cut marks on the mandible. (a) Posterior view of the right mandibular ramus in Hallazgo 7 (trauma numbers 1–2). (b) Posterior view of the right mandibular ramus in Hallazgo 9 (trauma numbers 3–5). (c) Lateral view of the left mandibular ramus in Hallazgo 6 (trauma numbers 6–8). (d) Lateral view of the left mandibular ramus in Hallazgo 7 (trauma numbers 9–10). (e) Posterior view of the left mandibular ramus in Hallazgo 9 (trauma numbers 11–14). (f) Inferior view of the mandibular body in Hallazgo 6 (trauma numbers 15–18). (g) Inferior view of the mandibular body in Hallazgo 7 (trauma numbers 19–21). (h) Inferior view of the mandibular bodies in Hallazgo 8 (trauma numbers 22–23).

Perimortem trauma was observed on four (Hallazgo 6, 7, 8, and 9) out of five mandibles (S4 Table). Parallel cut marks ranging in size from 1.1–5.4 mm in the coronal and sagittal directions are located on the posterior ascending ramus of the mandible and gonion of three individuals (Hallazgo 6, 7, and 9) (Fig 2A–2E). Moreover, clusters of incisions resulting from multiple slicing strokes in the coronal direction are concentrated on the inferior margin of the mandibular body of three individuals (Hallazgo 6, 7, and 8) (Fig 2F–2H). All of these markings were likely caused by slices that started from the posterior side and moved toward the anterior side, because of the aggregation of cut marks on the posterior side of the mandible.

**Fig 3:**
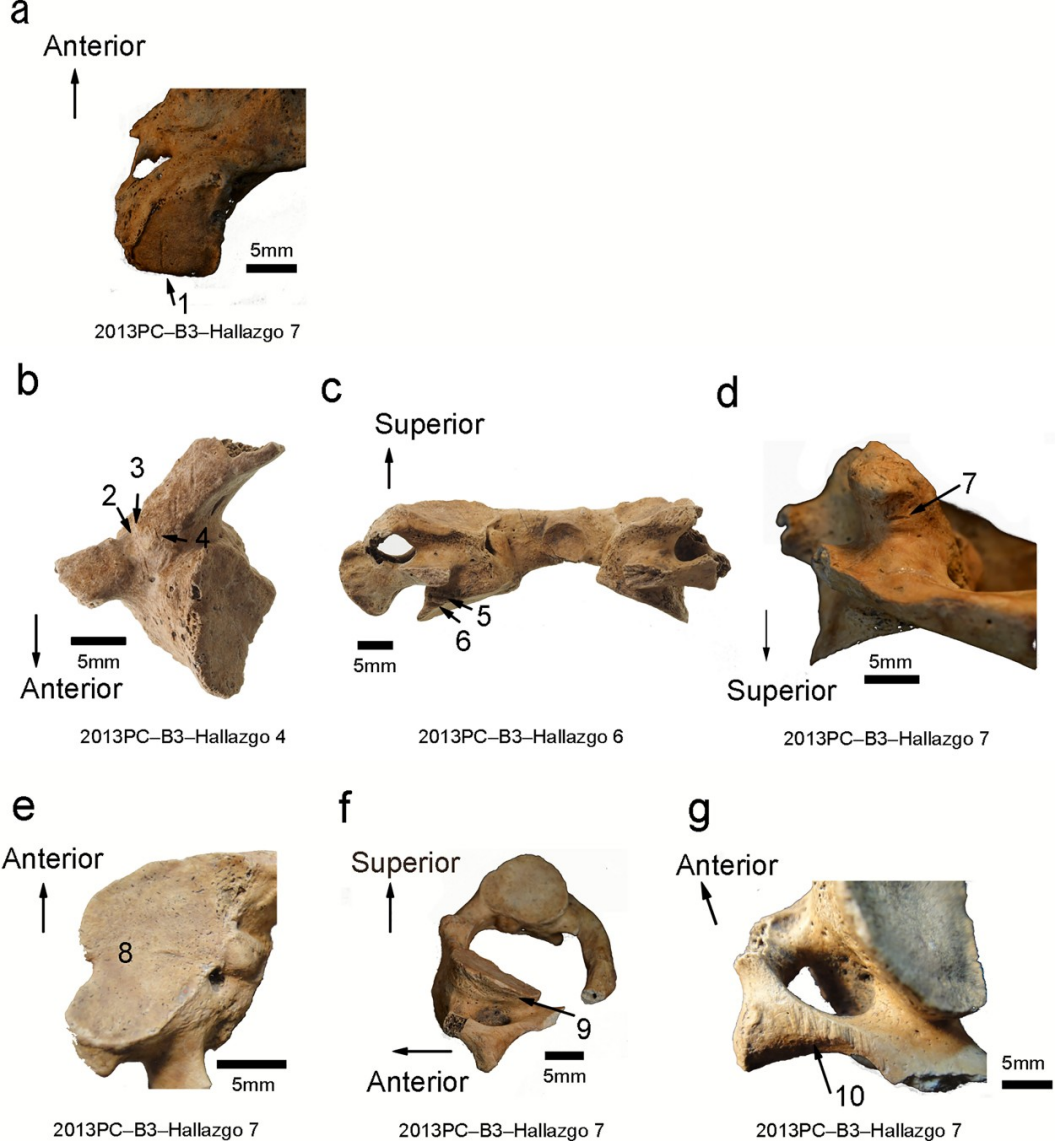
Macroscopic observation of cut marks on the occipital bone and atlas. (a) Inferior view of the right occipital condyle in Hallazgo 7 (trauma number 1). (b) Inferior view of the left inferior articular process of the atlas in Hallazgo 4 (trauma numbers 2–4). (c) Posterior view of the left inferior articular process of the atlas in Hallazgo 6 (trauma numbers 5–6). (d) Posterior view of the right inferior articular process of the atlas in Hallazgo 7 (trauma number 7). (e) Inferior view of the right inferior articular process of the atlas in Hallazgo 7 (trauma number 8). (f) Superolateral view of the left inferior articular process of the atlas in Hallazgo 7 (trauma number 9). (g) Posterosuperior view of the right transverse process of the atlas in Hallazgo 7 (trauma number 10).

Cut marks were also detected on one (Hallazgo 7) of the five occipital bones and on three (Hallazgo 4, 6, and 7) of the four atlases (S4 Table). The occipital bone of one individual (Hallazgo 7) has a cut mark on the right occipital condyle in the sagittal direction (Fig 3A); the direction indicates that force was applied from the inferior side. Regarding the atlas, Hallazgo 4 exhibits three cut marks parallel to each other on the left posterior side of the vertebral arch, which range in length from 1.6–5.4 mm in the superomedial-inferolateral direction (trauma numbers 2–4 of Fig 3B). Hallazgo 6 has two parallel cut marks, 5.8 mm and 7.0 mm in length, in the coronal direction on the posterior base of the left inferior articular process (Fig 3C). Hallazgo 7 exhibits a straight 1.8 mm cut mark on the right inferior articular process in the coronal direction (Fig 3), and the posterior base of the left superior articular process has a straight cut mark 1.7 mm in length in the coronal direction (Fig 3E); the left superior articular process reveals a 7.7 mm straight cut mark in the sagittal direction on the lateral side of the process (Fig 3F); and there is a cluster of incisions resulting from multiple slicing strokes on the superior posterior corner of the left transverse process adjacent to the vertebral artery (Fig 3G).

**Fig 4:**
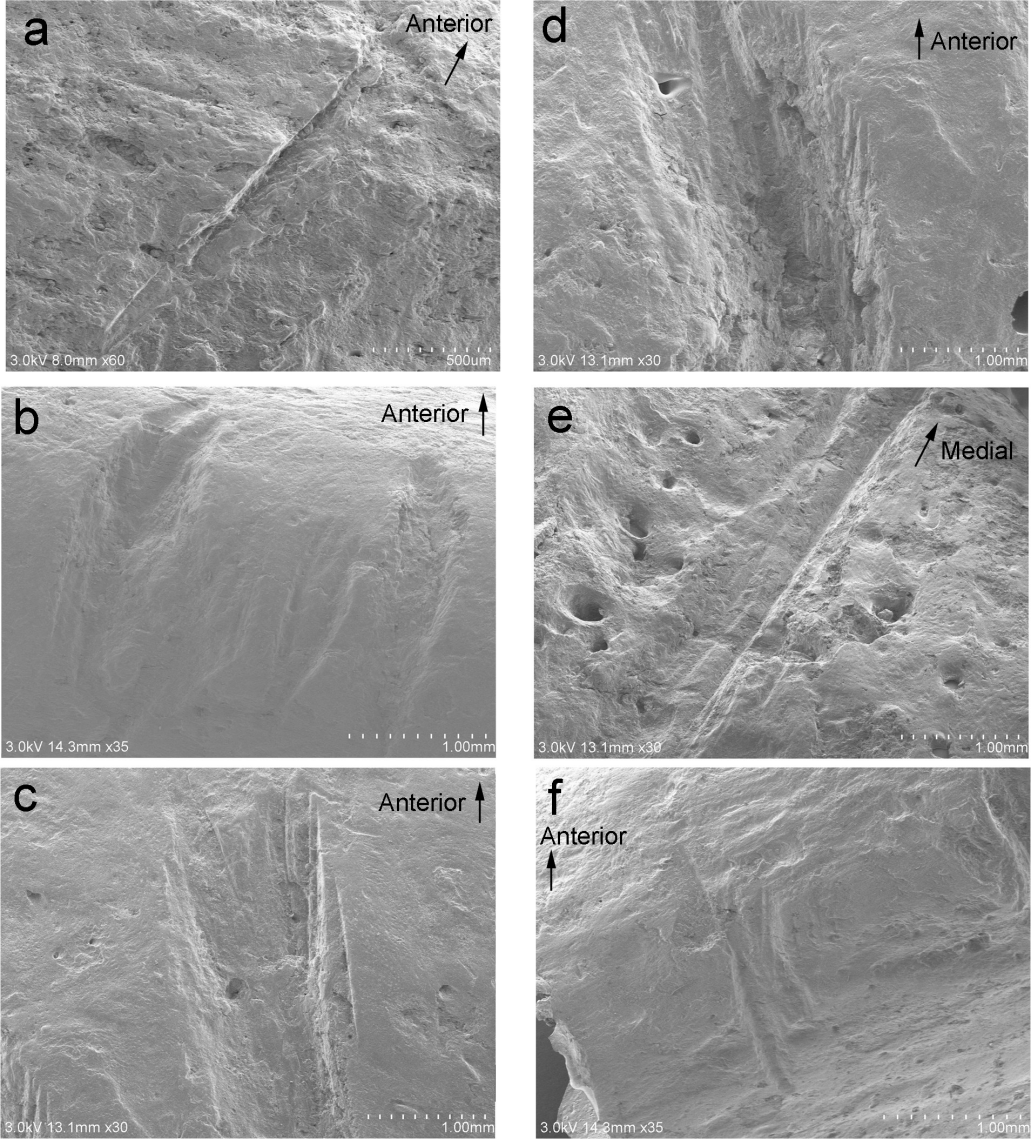
Microscopic observation of cut marks. (a) Microscopic magnification of the cut mark of the left inferior articular process of the atlas in Hallazgo 4 (trauma number 3 of Fig 3B). (b) Microscopic magnification of the cut mark of the left mandibular ramus in Hallazgo 6 (trauma number 6 of Fig 2C). (c) Microscopic magnification of the cut mark of the left mandibular ramus in Hallazgo 7 (trauma number 9 of Fig 2D). (d) Microscopic magnification of the cut mark of the left mandibular ramus in Hallazgo 7 (trauma number 10 of Fig 2D). (e) Microscopic magnification of the cut mark of the right inferior articular process of the atlas in Hallazgo 7 (trauma number 7 of Fig 3D). (f) Microscopic magnification of the cut mark of the left mandibular ramus in Hallazgo 9 (trauma number 13 of Fig 2E).

The microscopic magnification of cut marks found on Hallazgo 6 (Fig 4B magnifies the trauma number of Fig 2C), Hallazgo 7 (Fig 4C–4E magnifies trauma numbers 9 and 10 of Fig 2D and number 7 of Fig 3D), and Hallazgo 9 (Fig 4F magnifies trauma number 13 of Fig 2E) all revealed wide U-shaped grooves in cross-section accompanied by parallel striations along or inside them. This observation is consistent with the diagnostic standards of human-induced cut marks caused by stone blades [41–43]. On the other hand, microscopic observation of the cut marks on Hallazgo 4 showed a narrower groove in an acute V-shape in cross-section without parallel striations (Fig 4A magnifies trauma number 3 of Fig 3B). This observation is in accordance with the diagnostic standards of human-induced cut marks, possibly inflicted with sharper blades (including obsidian or metal blades) [38–40], although archaeological remains corresponding to such tools have not been recovered at the site. The width of the cut marks found on Hallazgo 6, 7, and 9 is similarly wider than that on Hallazgo 4, implying that similar tools were possibly used for decapitation in the former three individuals but not in the latter one individual.

These cut marks were almost the same for each individual in terms of their direction, length, and shape. The distribution and direction of the cuts suggests blades were inserted from the latero-posterior part of the neck and moved transversally. The lack of signs of healing indicates that these five individuals were cut perimortem either before death or just after. These individuals do not display any differences in color between the modified and original bone surfaces, suggesting that the damage to the crania was not recent. At the time of excavation, the crania, mandibles, and cervical vertebrae remained articulated; therefore, the bodied had been processed while still fleshed.

### Antemortem trauma

**Fig 5:**
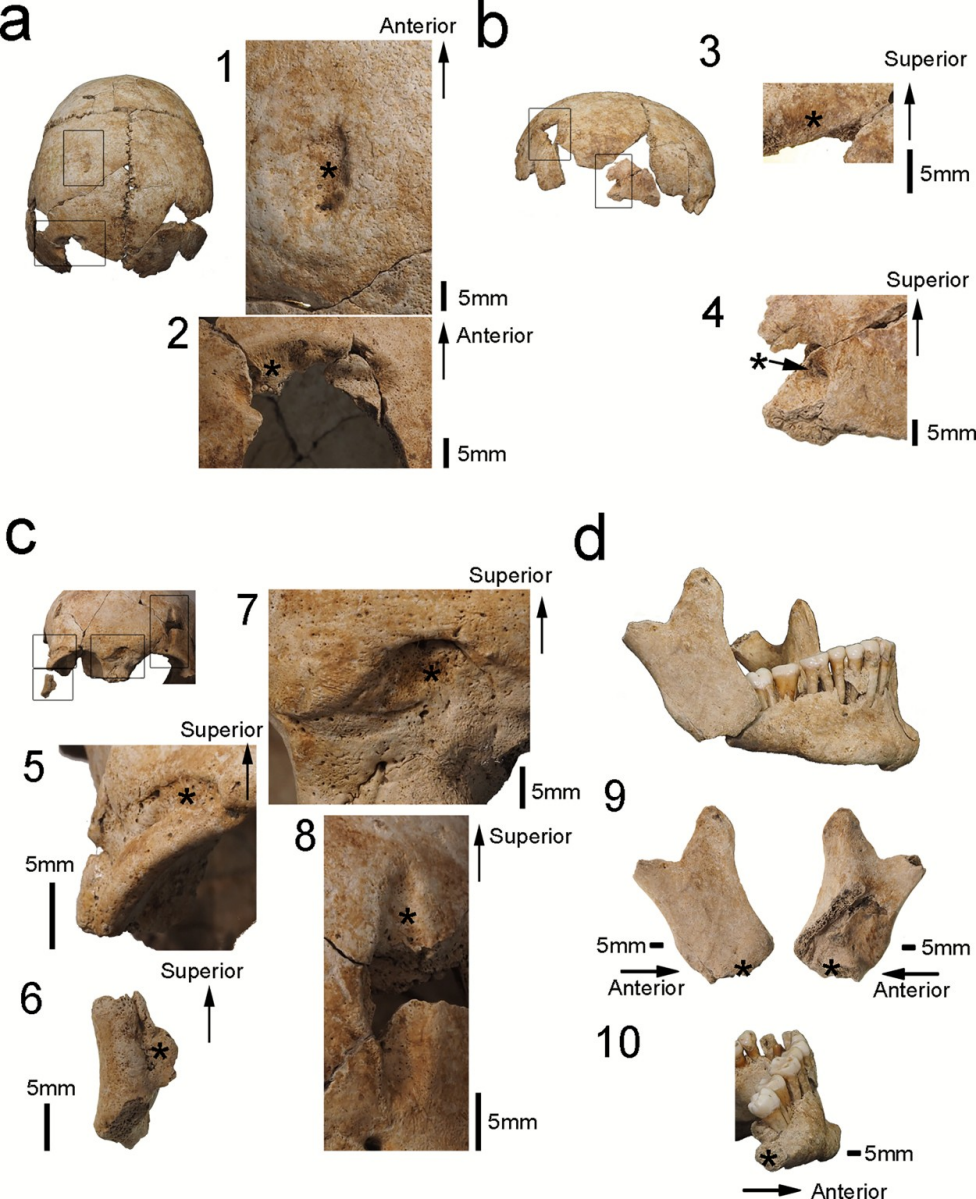
Healed fractures in Hallazgo 12. Two depressed fractures on the left parietal bone (trauma numbers 1–2) (superior view of the cranium); two depressed fractures on the right parietal bone (trauma numbers 3–4) (right lateral view of the cranium); four depressed fractures on the frontal and right zygomatic bones (trauma numbers 5–8) (anterior view of the cranium); formation of pseudoarthrosis observed between the right mandibular ramus (trauma number 9) and mandibular body (trauma number 10). * shows the location of the trauma.

Among all six isolated crania, antemortem trauma was detected on only Hallazgo 12 from the Early Cajamarca Period (S5 Table). We detected four depressed fractures on the right and left parietal bones (trauma numbers 1, 2, 3, and 4 of Fig 5A and 5B), three depressed fractures on the frontal bone (trauma numbers 5, 7, and 8 of Fig 5C), and one depressed fracture on the right zygomatic bone (trauma number 6 of Fig 5C). Most of the fractures have an elliptical shape, with a long diameter of almost 1–2 cm, and their depth is limited to the outer table or diploae (trauma numbers 1, 2, 5, 7, and 8 of Fig 5A–5C). Additionally, the medial half of the right zygomatic bone was depressed compared to the lateral half (Fig 5C). These elliptical depressed fractures observed on this individual are similar in terms of shape, size, and depth, and were classified as blunt force fractures produced by the direct application of force, possibly over the course of multiple incidences.

The only case of pseudoarthrosis in this individual is that of the mandible (Fig 5D). This fracture is severe, involving the right mandibular body and ramus, both of which were detached and healed without fusion (trauma numbers 9 and 10 of Fig 5D). The fracture was also classified as a blunt force fracture, produced by the direct application of force to the mandible in the lateral direction.

In all cases, the presence of a healing reaction in the remodeling phase, with the formation of smooth edges around the fractures, shows that the fractures were antemortem; the individual survived after suffering the fractures. Although Hallazgo 12 lacks perimortem trauma on the cranium and mandible possibly due to poor preservation, the cranium and mandible were articulated when excavated. This suggests that the fresh body was possibly decapitated, while a decapitated head associated with healed and healing fractures indicates that this individual was involved in multiple incidences of violence during his lifetime.

## Discussion

### Systematic postmortem modification patterns associated with decapitation

The six crania excavated from the sunken court represent the first evidence for decapitation at Pacopampa, as the 103 individuals examined from the two preceding cultural phases, the Middle and earlier half of the Late Formative Periods, did not exhibit any signs of perimortem trauma [35]. Four out of six crania (Hallazgo 6, 7, 8, and 9) that were excavated in the Early-Cajamarca-Period layer indicated the calibrated ^14^C ages of the latter half of the Late Formative Period and the Final Formative Period. The location and direction of their cut marks were strikingly similar among the decapitated individuals from this time period: most were perpendicular to the posterior ascending ramus of the mandible, and the posterior aspect of the atlas is suggestive of cut marks made from behind the individual in an attempt to detach the head from the body. Additionally, the cut marks on the articular surfaces of the occipital bone and atlas suggest that the joints of the atlas were severed. The presence of incisions resulting from multiple slicing strokes on the transverse process of the atlas further suggests that the individuals would have suffered lethal cuts to the vertebral artery and spinal cord if alive when the cuts were made. Microscopic observation reveals similarities in cut mark morphology among four isolated crania, indicating that the stone tools used for the decapitations were likely similar, while the microscopic observation of the cut marks on Hallazgo 4 shows a narrower groove in an acute V-shape in the cross-section view. The fact that the four isolated crania (Hallazgo 6, 7, 8, and 9) have a similar cut mark morphology and radiocarbon date suggests that they were modified at the same time. However, the presence of a sharper cut mark in the one cranium (Hallazgo 4) further suggests that it was decapitated by a different tool possibly at a different time. If the sharper cut mark in that cranium was made definitely at the different time, it is likely that there were multiple decapitators.

Among the non-biased sex ratio of the skeletal population at Pacopampa [38], the decapitated remains were all discovered to be young to middle-aged males, suggesting that this demographic was preferred for decapitation. At the Huaca de la Luna of the Moche society on the northern coast, it was detected that the trophy heads were a highly selected sample of individuals, as indicated by a complete lack of females, children, and elderly adults [21]; this is similar to the demographic of the isolated crania at Pacopampa. The similarities in cut-mark distribution, direction, and shape, as well as the idea of selected individuals, implies that the isolated crania were carefully prepared using a fixed method and those who modified the heads may have been professional decapitators.

These cut marks at Pacopampa, predominantly located on the posterior section of mandibles and cervical vertebrae, differ from the general cut-mark patterns found at Moche, where some cut marks were aggregated on the anterior section of cervical vertebrae across the victims’ throats [16, 21]. The body treatment observed at Moche parallels an iconographic depiction of the throat-slitting and blood-letting of bound males and the drinking of their blood [19]. Violent torture preceding death, associated with considerable pain and bleeding, may have contributed to the spectacle of Moche’s ritualistic performance [49]. In addition, the microscopic observations of most cut marks at Pacopampa revealed grooves with several apices, judged to have been made by stone tools; this is in contrast to most of the cut-mark profiles at Moche, which are sharp V-shaped grooves with straight walls, a morphological pattern consistent with those created using a copper blade (the tumi depicted in Moche iconography and actually excavated from the Dos Cabezas site at Moche) [14, 49, 50].

The Pacopampa remains lack drilled holes in the superior of the cranium used to accommodate a carrying cord, which was a pattern standardized by the Nasca and Wari, where individuals were modified postmortem into trophy heads [17, 25, 51]. The Nasca trophy heads were disembodied by sharp obsidian knives, the foramen magnum was enlarged for brain extraction, and holes were drilled into the foreheads to insert carrying cords [52]. The positions and sizes of the perforations and the enlargements of the foramen magnum of skull found in Nasca vary significantly [17]. In contrast, at Wari, these methods of trophy-head creation were more standardized through the use of uniform perforations of size, shape, and location [17]. The isolated crania excavated at Pacopampa do not exhibit the enlargement of foramen magnum or perforation of the frontal bone, which are two diagnostic features of trophy heads [53]. However, this only indicates that they were not suspended for display and could still represent trophy heads. It is possible that a distinct pattern might have developed at Pacopampa in the early stages of ritual offerings of human skulls in terms of decapitation technique, procedures, and display.

### Emergence of decapitation at Pacopampa

Ritual violent behaviors at Pacopampa may have been related to the emergence of social stratification in the Late Formative Period [38]. The high value objects found in elite graves and their special burial treatment demonstrated that social inequality at Pacopampa could date back to the beginning of the Late Formative Period [39]. Violence may have functioned to maintain the hierarchical structure of societies and the prestige of the ruling elite through ritualized killing meant to reflect the elite’s monopoly over life, death, and socioeconomics [4]. The portrayal of humans with fierce animal features found at the temple of Chavín de Huántar may have represented the incorporation of natural power into human forms [54]. The precedent study suggested that these figures might have exercised fierce forces on victims during ritual practices, such as in the temple of Chavín de Huántar [54]. Anthropomorphized creatures depicted on vessels, stone sculptures, and wall reliefs at Pacopampa were detected to be from the Middle and Late Formative Periods, previous to the emergence of decapitation. During the Middle and Late Formative Periods, when complex societies may have first appeared and developed, the severe but healed cases of trauma concentrated on crania that lacked defensive wounds appear to have been a result of fierce forces applied under controlled conditions at Pacopampa [38].

In this study, the radiocarbon date of four isolated crania is from the latter half of the Late–Final Formative Periods, when the sunken court that functioned as a part of a ceremonial center was abandoned, while the stratigraphy and archaeological artifacts (e.g., kaolin vessels) indicate that these ritual activities belong to the Early Cajamarca Period. The court ceased functioning in the Final Formative Period, but the ruins continued to be a place of rituals during the Early Cajamarca Period. The presence of perimortem trauma in the isolated crania suggests an increased variability in violence at Pacopampa, although little is known about the sociopolitical backgrounds of that time.

The question of whether the isolated crania belonged to men who were members of the community or to captured foreign enemy combatants is important, as it sheds light on the motivation of such practices. At Pacopampa, decapitation was continually observed both in the latter half of the Late–Final Formative Periods and the Early Cajamarca Period. However, the first known remains did not have healed fractures, and those who were transformed into trophies were at least not likely to have been involved in severe violence in the latter half of the Late–Final Formative Periods. The lack of defensive architecture, fortification, and property destruction at Pacopampa suggests a lack of the presence of organized battles in the Formative Period [38], and distribution of individuals with trauma was concentrated in ritual areas of the third platform; decapitation is thus considered to have represented ritual activities and not warfare-related violence, at least at the beginning of the practice. An excavation at the ceremonial complex of Chankillo in the coastal desert uncovered ceramic warrior figurines holding weapons and in combat position, which suggests conflict underlying the collapse of the Chavín culture in the Final Formative Period [55]. This is consistent with the changes over time at Pacopampa towards involvement in severe violence. Therefore, the decapitated individuals may have been connected to the social disturbance in the Final Formative Period.

Why the Formative-Period heads were discovered in the Cajamarca layer might be explained by assuming that bodies that were already deceased were used in the rituals, as seen in the Middle Sicán (AD 900–1100), where prolonged curation of dead bodies was carried out [56]. Documentation by the Spaniards also indicates that the preserved body parts of Inca lords were used in ancestor veneration rituals [57]. If the prolonged curation of dead bodies explains the specific treatment of trophies, three interpretations are possible for the preservation of the isolated crania with articulation: (1) they were kept indoors as mummies and were repeatedly used in rituals; (2) they were placed in situ after rituals, and subsequent ritual activities were done in the same place; or (3) they were related to the act of destruction of an already deceased mummified ancestor. With the current data, however, it remains unknown which interpretation is more plausible for the given situation and how the isolated crania were preserved with articulation. There is no evidence that they were wrapped in textiles. Another but less plausible interpretation is that the isolated crania were transported as ritual offerings from the coast. The Pacopampa individuals seem likely to have consumed low amounts of maritime resources, such as fish, shellfish, and marine mammals, as the low nitrogen isotope ratio of the six isolated crania indicates that maritime resources were seldom part of their diets (see S1 Text; S1 Fig; S6 Table). Furthermore, the carbon and nitrogen isotopic ratios of the six isolated crania from the Pacopampa site were compared with those from the neighboring Kuntur Wasi site at the Kuntur Wasi (800–550BC), Copa (550–250 BC), and Sotera (250–50 BC) phases (S1 Text; S1 Fig). Although the Pacopampa site had significantly higher carbon isotopic ratios than the Kuntur Wasi (P = 0.001) and Copa (P = 0.021) phases of the Kuntur Wasi site, the overlapped isotopic ratios of the Pacopampa site and the Kuntur Wasi’s Sotera phase (P = 0.794) suggest that the decapitated individuals had consumed similar foods as did the local people in the Kuntur Wasi site during the same period. (S1 Text; S1 Fig). This supports the theory that the already deceased bodies were repeatedly used in ancestor veneration rituals.

Ritual offerings of human skulls were practiced at Pacopampa, as has also been seen in the Nasca, Moche, and Wari societies. However, it is not known whether the practices at Pacopampa were associated with the offerings of the life of a living being to the supernatural. Even though it is possible that the Pacopampa decapitation did not represent human sacrifice, the isolated crania in and of themselves might have expressed sacredness in a ritual setting [28]. The isolated crania are the first to exhibit cut marks at Pacopampa, suggesting that a different form of violence that originated in rituals may have existed during the latter half of the Late–Final Formative Periods. This study offers indisputable bioarchaeological evidence of ritual offerings of human skulls (regardless of supplication of bodies that were already deceased or not), which confirms the reality of a contemporaneous iconographic motif of decapitation and extends the chronology of the practices back to the Formative Period in the northern Peruvian highlands.

## Supporting information

S1 Text Supporting information on carbon and nitrogen isotope ratios at Pacopampa. (DOC)

S1 Fig Carbon and nitrogen isotope ratios at Pacopampa in comparison with food resources. Ellipses suggesting food resources’ isotope ratios estimated from the data of the precedent studies. Furthermore, the carbon and nitrogen isotopic ratios of the isolated crania from the Pacopampa site were compared with those from the neighboring Kuntur Wasi site at the Kuntur Wasi (800–550BC), Copa (550–250 BC), and Sotera (250–50 BC) phases. Data were corrected isotopic fractionation.
(TIF)

S1 Table Age at death and sex composition of Pacopampa. (XLS)

S2 Table Calibrated radiocarbon date at Pacopampa. (XLS)

S3 Table Diagnoses of human skeletons at Pacopampa. (XLS)

S4 Table Perimortem trauma at Pacopampa. (XLS)

S5 Table Antemortem trauma at Pacopampa. (XLS)

S6 Table Raw carbon and nitrogen isotope data at Pacopampa. (XLSX)
