# Supplementary material for: Bioarchaeological evidence of decapitation from Pacopampa in the northern Peruvian highlands
Source: PLoS One. 2019 Jan 8;14(1):e0210458. doi: 10.1371/journal.pone.0210458 (PMC6324785; doi:10.1371/journal.pone.0210458)
Supplement: S1 Text — (DOC) [file pone.0210458.s001.doc]

**Supporting information on carbon and nitrogen isotope ratios at Pacopampa**

**Materials and Methods**

The subsistence of six isolated crania was estimated to explore whether they were belonged to foreign enemy combatants. Data for comparison were derived from the neighboring Kuntur Wasi site in the northern highlands [1] (personal communication with Y. Seki and M. Yoneda [15 October 2018]). The Kuntur Wasi human remains belonged to three different cultural phases: (1) the former half of the Late Formative Period (Kuntur Wasi phase, 800–550BC); (2) the latter half of the Late Formative Period (Copa phase, 550–250 BC); and (3) the Final Formative Period (Sotera phase, 250–50 BC).

To measure carbon and nitrogen isotope ratios, gelatin collagen was extracted from bone specimens [2, 3]. After exotic organic substances, such as humic acid, were eliminated by soaking the bone specimens in 0.2M NaOH, the bone mineral portions were removed through decalcification with 1.2M HCl. Only gelatin collagen was extracted from the remaining organic portions by heating them to 90°C for 12 hours. The isotope values were measured using an elemental analyzer coupled to an isotope ratio mass spectrometry (Flash 2000 and Delta V Advantage, Thermo Fisher Scientific, Bremen, Germany) at the University of Tokyo in Tokyo, Japan. The standard deviation was 0.1‰ for δ^13^C and 0.3‰ for δ^15^N. The state of the collagen preservation with the C/N ratio was assessed, and because fresh bone collagen ranges between 2.9 and 3.6 [4], it is possible that the collagen with an unacceptable C/N ratio had been subjected to alteration or pollution. Data were further corrected for isotopic fractionation between ingested food and bone collagen using 4‰ for δ^13^C value and 3‰ for δ^15^N value. We corrected for the Suess effect with +1.5‰ for carbon isotope ratios of modern foods. Food resources’ isotope ratios were estimated from the data of the precedent studies [5–11]. One-way analysis of variance with post-hoc Tukey HSD (honest significant difference) test was calculated for differences between Pacopampa and Kuntur Wasi. The statistical analyses were computed by the statistical package IBM SPSS Statistics 22.

**Results**

All of the gelatin collagen C/N ratios from the six individuals were within the acceptable range, suggesting that the collagens were not subjected to diagenetic alteration (S6 Table). The individuals are likely to have consumed C_3_ and C_4_ plants, such as manioc, beans, potato, arracacha, and maize, and terrestrial animals (S1 Fig). Although the Hallazgo 8 individual revealed evidence of slightly higher nitrogen isotope ratios, it is possible that the individual consumed more terrestrial animals than the others. However, the dietary variation among the six isolated crania was not substantial, and marine resource consumption was rare. The decapitated individuals from the Pacopampa site had significantly higher carbon isotopic ratios than the individuals from the Kuntur Wasi (P=0.001) and Copa (P=0.021) phases of the Kuntur Wasi site, while there is no significant difference in the nitrogen isotopic ratios among groups (P=0.297). Although we cannot detect any significant difference in the carbon isotopic ratios between the Pacopampa site and the Kuntur Wasi’s Sotera phase (P=0.794) due to the small sample size, the overlapped isotopic ratios of two groups suggest that the decapitated individuals consumed similar foods as did the local people in the Kuntur Wasi site during the same period (S1 Fig).

**References**

1. Seki Y, Yoneda M. Reconstruction of the dietary patterns in the Formative Period of the north highlands of Peru: consideration by stable carbon and nitrogen isotope analysis. Bulletin of the National Museum of Ethnology. 2004; 28: 515-537 (in Japanese with English summary). Japan.

2. Longin R. New method of collagen extraction for radiocarbon dating. Nature. 1971; 230: 241–242.

3. Yoneda M, Tanaka A, Shibata Y, Morita M, Uzawa K, Hirota M, Uchida M. Radiocarbon marine reservoir effect in human remains from the Kitakogane Site, Hokkaido, Japan. J Archaeol Sci. 2002; 29: 529–536.

4. DeNiro MJ. Postmortem preservation and alteration of in vivo bone collagen isotope ratios in relation to palaeodietary reconstruction. Nature. 1985; 317: 806–809.

5. DeNiro MJ. Marine food sources for prehistoric coastal Peruvian camelids: isotopic evidence and implications. In: Wing ES, Wheeler JC, editors. Economic Prehistory of the Central Andes (BAR international series). Oxford: British Archaeological Reports; 1988. pp. 119–129.

6. DeNiro MJ, Hastorf CA. Alteration of ^15^N/^14^N and ^13^C/^12^C ratios of plant matter during the initial stages of diagenesis: Studies utilizing archaeological specimens from Peru. Geochim Cosmochim Acta. 1985; 49: 97–115.

7. Falabella F, Planella MT, Aspillaga E, Sanhueza L, Tykot RH. Dieta en sociedades alfareras de Chile central: aporte de análisis de isótopos estables. Chungara. 2007; 39: 5–27. Chile.

8. Szpak P, White CD, Longstaffe FJ, Millaire JF, Sanchez VFV. Carbon and nitrogen isotopic survey of northern Peruvian plants: Baselines for paleodietary 44 and paleoecological studies. Plos One. 2013; 8: e53763.

9. Tieszen LL, Chapman M. Carbon and nitrogen isotopic status of the major marine and terrestrial resources in the Atacama desert of northern Chile. In: Aufderheide A, editor. Proceedings of the First World Congress on Mummy Studies. Tenerife: Museo Argueológico y Ethnográfico de Tenerife; 1992. pp. 409-425. Spain.

10. Turner BL, Kingston JD, Armelagos GJ. Variation in dietary histories among the immigrants of Machu Picchu: Carbon and nitrogen isotope evidence. Chungara. 2010; 42: 515-534. Chile.

11. Verano JW, DeNiro MJ. Locals or foreigners? Morphological, biometric and isotopic approaches to the question of group affinity in human skeletal remains recovered from unusual archaeological contexts. In: Sandford MK, editor. Investigations of Ancient Human Tissue: Chemical Analysis in Anthropology. Langhorne: Gordon & Breach Science Publishers; 2003. pp. 361–386.
