# Supplementary figures and images for: Bioarchaeological evidence of decapitation from Pacopampa in the northern Peruvian highlands
Source: PLoS One. 2019 Jan 8;14(1):e0210458. doi: 10.1371/journal.pone.0210458 (PMC6324785; doi:10.1371/journal.pone.0210458)

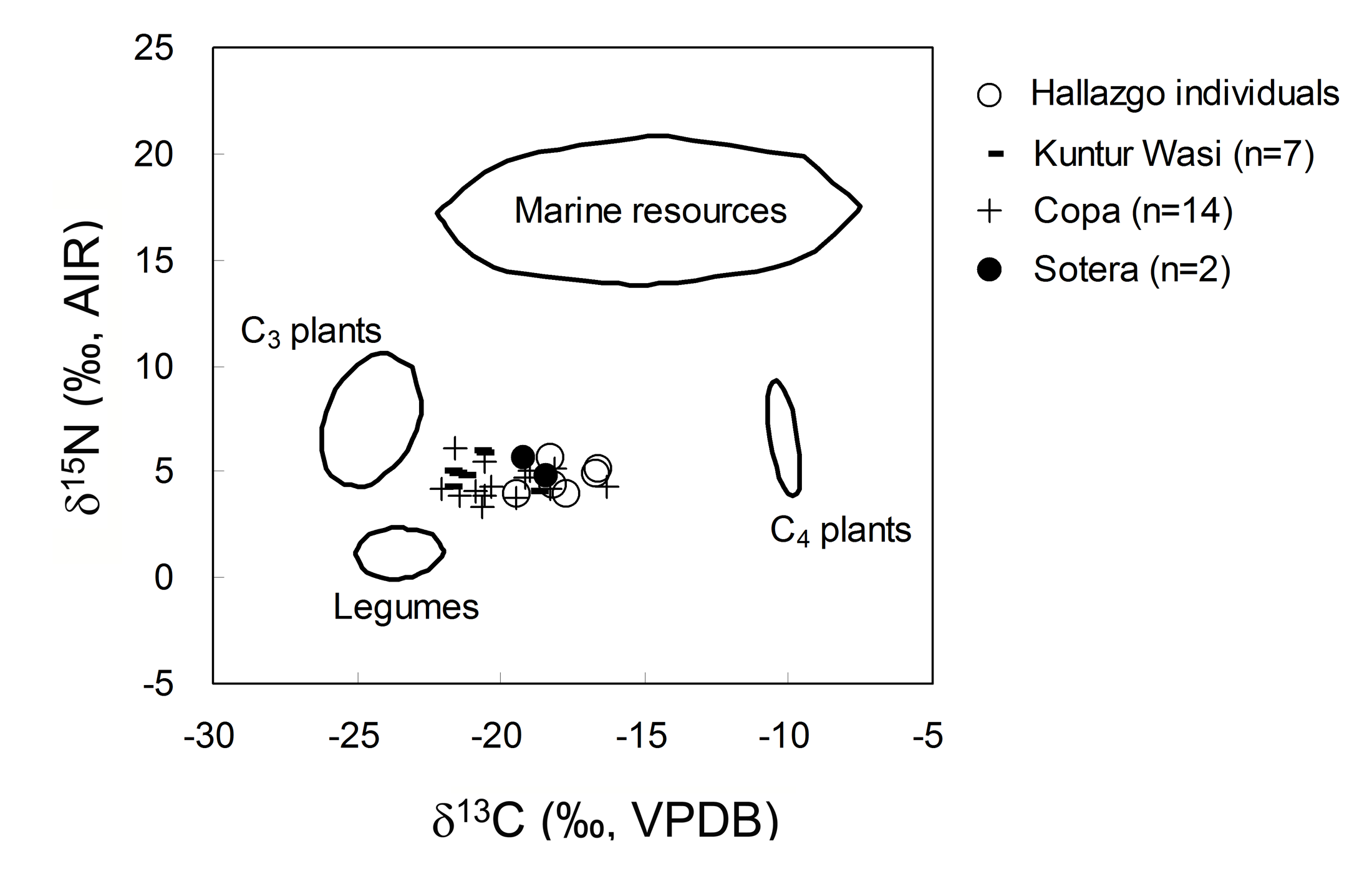

Supplement: S1 Fig — Ellipses suggesting food resources’ isotope ratios estimated from the data of the precedent studies. Furthermore, the carbon and nitrogen isotopic ratios of the isolated crania from the Pacopampa site were compared with those from the neighboring Kuntur Wasi site at the Kuntur Wasi (800–550BC), Copa (550–250 BC), and Sotera (250–50 BC) phases. Data were corrected isotopic fractionation. (TIF) [file pone.0210458.s002.tif]
